# Generation of hexagonal close-packed ring-shaped structures using an optical vortex

**DOI:** 10.1515/nanoph-2021-0437

**Published:** 2021-10-20

**Authors:** Haruki Kawaguchi, Kei Umesato, Kanta Takahashi, Keisaku Yamane, Ryuji Morita, Ken-ichi Yuyama, Satoyuki Kawano, Katsuhiko Miyamoto, Michinari Kohri, Takashige Omatsu

**Affiliations:** Graduate School of Engineering, Chiba University, 1-33 Yayoi-cho, Inage-ku, Chiba 263-8522, Japan; Department of Applied Physics, Hokkaido University, Kita-13, Nishi-8, Kita-ku, Sapporo 060-8628, Japan; Department of Chemistry, Osaka City University, 3-3-138 Sugimoto Sumiyoshi-ku, Osaka 558-8585, Japan; Graduate School of Engineering Science, Osaka University, 1-3 Machikaneyama, Toyonaka, Osaka 560-8531, Japan; Molecular Chirality Research Center, Chiba University, 1-33 Yayoi-cho, Inage-ku, Chiba 263-8522, Japan

**Keywords:** laser induced forward transfer, nanostructures, optical vortex, orbital angular momentum, singular optics, structural colors

## Abstract

An optical vortex possesses a ring-shaped spatial profile and orbital angular momentum (OAM) owing to its helical wavefront. This form of structured light has garnered significant attention in recent years, and it has enabled new investigations in fundamental physics and applications. One such exciting application is laser-based material transfer for nano-/micro-fabrication. In this work, we demonstrate the application of a single-pulse optical vortex laser beam for direct printing of ring-shaped structures composed of hexagonal close-packed, mono-/multi-layered nanoparticles which exhibit ‘structural color’. We compare and contrast the interaction of the vortex beam with both dielectric and metallic nanoparticles and offer physical insight into how the OAM of vortex beams interacts with matter. The demonstrated technique holds promise for not only photonic-based nano-/micro-fabrication, but also as a means of sorting particles on the nanoscale, a technology which we term ‘optical vortex nanoparticle sorting’.

## 1 Introduction

Laser-induced forward transfer technologies (LIFT) [1, 2] make use of laser beams to project material onto a receiver substrate. This technique is noncontact, does away with nozzles (and avoids issues such as clogging), and it is capable of printing a variety of shapes and patterns using micrometer-scale liquid droplets. As such, it has found application in fields including printed photonics/electronics/spintronics and integrated optical circuits. To date, most laser-induced forward transfer studies have been performed using conventional Gaussian beams with a planar wavefront.

An optical vortex laser beam possesses a helical wavefront and a ring-shaped spatial profile featuring a dark central spot and orbital angular momentum (OAM) with a characteristic topological charge ℓ [3, 4]. This type of laser beam has been applied to the investigation of a variety of new fundamental physics, and applications at the micro-/nano-scale, including optical trapping and manipulation [5], optical/quantum free-space/fiber telecommunications [6], [7], [8], and super-resolution microscopy, with spatial resolution beyond the diffraction limit [9, 10]. Importantly, optical vortex beams have led to innovations in advanced laser materials processing, wherein the optical vortex is able to transfer OAM to the target material and generate helical nanostructures [11], [12], [13], [14], [15].

In recent years, we and our coworkers have discovered that a single optical vortex pulse is capable of twisting an irradiated donor material (including materials with high viscosity), and eject it, propelling a picoliter (pL)-scale spinning microdroplet of the material with an extremely long flight distance [16]. This vortex laser-induced forward transfer mechanism represents a new physical manifestation of the interaction between the OAM of light and matter, and as a technique, it offers a new approach to the direct-print of ultrahigh viscosity donor materials with ultrahigh spatial resolution.

**Figure 1:**
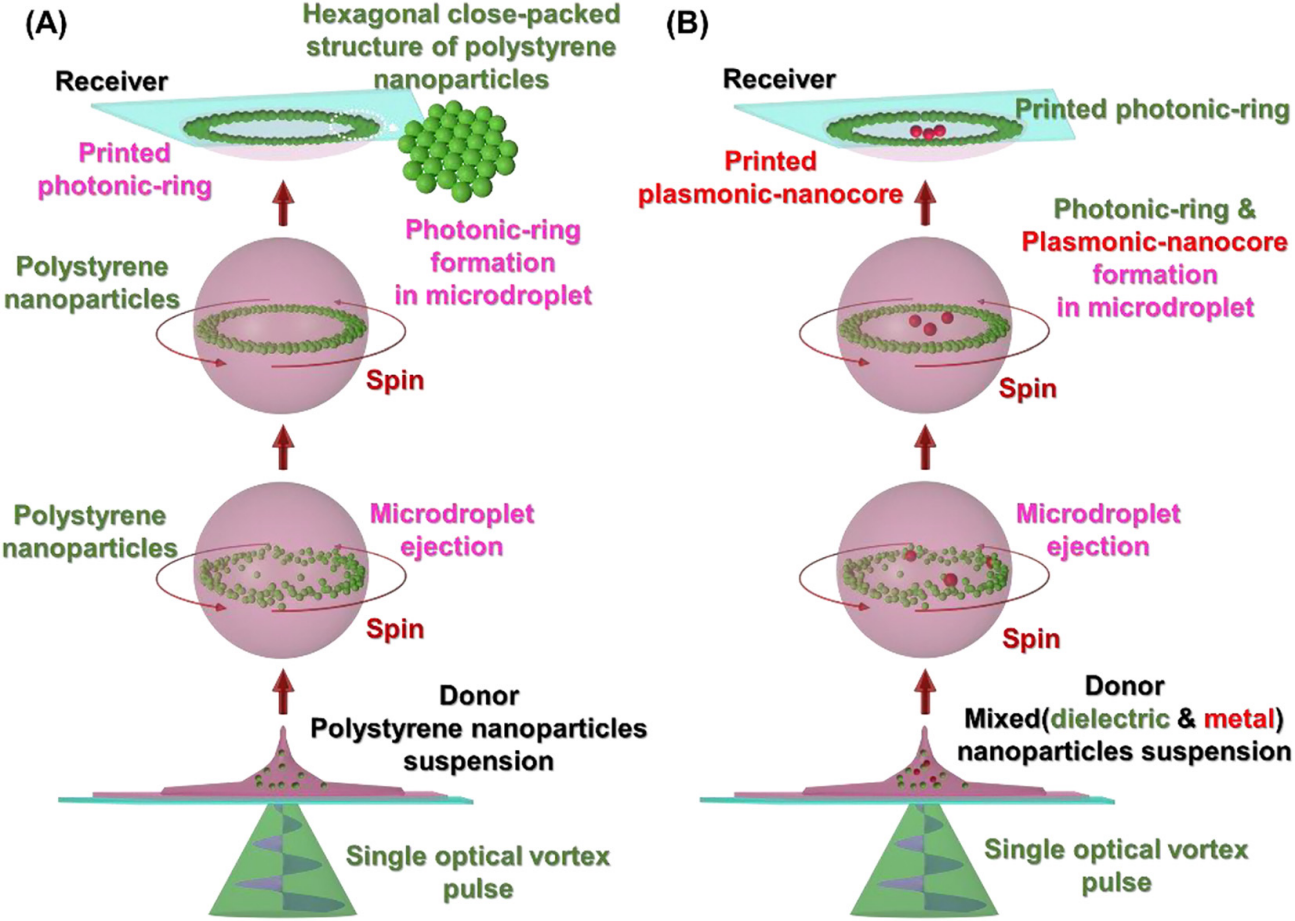
(A) Schematic diagram of the direct print of a photonic-ring (hexagonal close-packed and monolayered ring) by illumination of a donor suspension by a single optical vortex pulse. (B) Basic concept for ‘optical vortex nanoparticle sorting’. Here, a dielectric photonic-ring and a metallic plasmonic nanocore are printed and spatially separated on a receiver simply by illumination by a single optical vortex pulse.

Extending beyond conventional LIFT technologies which make use of Gaussian laser beams, and our previous application of a vortex laser beam, here, we fabricate ring-shaped structures composed of hexagonal close-packed, mono-/multi-layered nanoparticles using a single-pulse vortex laser beam-based LIFT technique. These ring-shaped structures are herein referred to as ‘photonic-rings’, and exhibit structural color which is characteristic of the nanoparticle size, packing density, and refractive index. A schematic diagram of the fabrication technique and physical process is shown in Figure 1(A). A single optical vortex pulse ejects a pL-scale spinning microdroplet from a colloidal suspension of dielectric nanoparticles. The dielectric nanoparticles in the microdroplet are then packed at the edge of the microdroplet. Subsequently, the microdroplet can be projected by the laser beam and printed to form a photonic-ring on a receiver substrate a long distance (millimeter-scale) away. To the best of our knowledge, such structured packing of nanoparticles inside a microdroplet and subsequent fabrication of photonic-rings has never been reported in all prior works regarding laser induced single microdroplet ejection.

The mechanism behind photonic-ring formation using an optical vortex is entirely different from the ‘coffee ring effect’ which manifests from Marangoni flow [17, 18]. The formation of photonic-rings is unique to the application of optical vortex beams and has never been observed using axisymmetrically polarized beams with a ring-shaped spatial profile and without OAM, or with the use of conventional Gaussian beams. In this work, we offer insight into the mechanism behind the formation of these photonic-rings and investigate the role that OAM plays. As a part of this investigation, we examine the interaction of OAM with dielectric and metallic nanoparticles and find that the mechanics differ significantly, so much so that a single optical vortex pulse has the potential to be applied as a means of spatially separating dielectric and metallic nanoparticles; a process which we term ‘optical vortex nanoparticle sorting’ which is shown schematically in Figure 1(B). Furthermore, we demonstrate the use of optical vortex-induced forward transfer to the direct-print of highly-localized, single gold (Au) nanoparticles onto a metallic-coated substrate. We believe that the placement of Au (and other metallic) nanoparticles with such high accuracy will have application within the field of plasmonics and facilitate the development of new structures such as ‘plasmonic-nanocores’.

Understanding the unique interaction between OAM of light and matter will pave the way toward development of advanced techniques for low-cost, high-efficiency fabrication of photonic-nanostructures [19], [20], [21]. Such photonic-nanostructures, which operate beyond the diffraction limit will facilitate exotic light–matter interactions leading to new technologies such as solar light harvesting systems [22, 23] and biomimetic structurally-colored materials [24].

## 2 Methods

**Figure 2:**
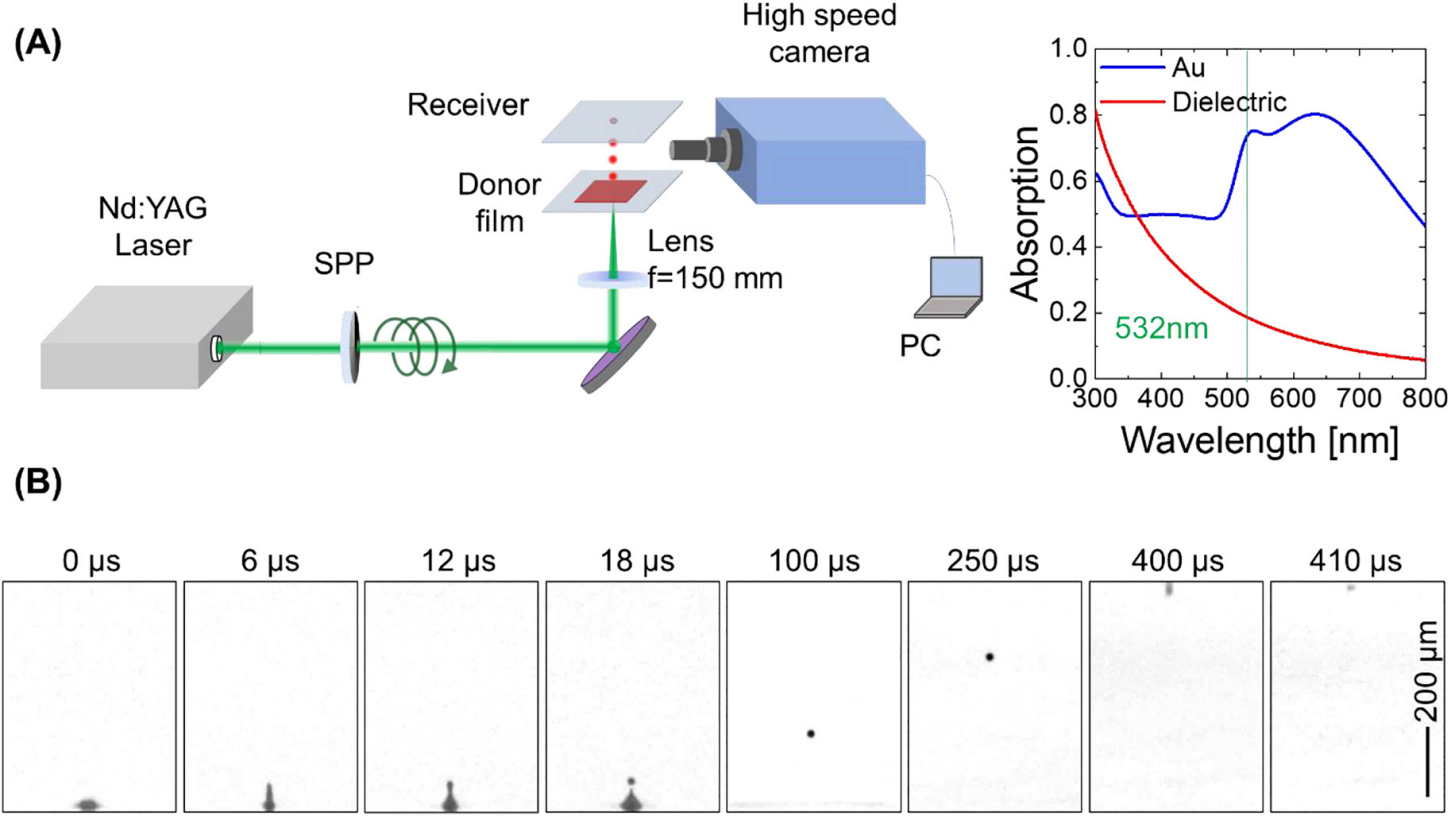
(A) Experimental setup. An inset shows the optical density (absorption) of polystyrene and Au nanoparticles in suspension. (B) Temporal evolution of the ejection of a single microdroplet from the donor liquid film.

The experimental setup is shown in Figure 2(A). The dielectric donor material used in this work comprised a colloidal aqueous suspension of monodisperse polystyrene nanoparticles (diameter: ca. 230 nm, refractive index: ca. 1.59, density: 1.57 wt%). These nanoparticles were coated with a ca. 2.5 nm thick polydopamine layer [25] and the suspension was prepared via emulsifier-free emulsion polymerization using hydrophilic comonomers. The optical density of the suspension was measured to be 0.19 at 532 nm (optical path: 10 mm), and the suspension was dropped on a glass substrate to form a liquid film with a thickness of ca. 40 µm. A linearly polarized, frequency-doubled Q-switched nanosecond Nd:YAG laser with a wavelength of 532 nm and a pulse duration of ca. 10 ns was employed as the laser source, and its output was converted to a linearly polarized 1st order optical vortex with ℓ = 1 by a spiral phase plate (SPP). The optical vortex was loosely focused to a ring-shaped spot with a diameter of ca. 50 µm onto the donor film, incident from the backside. All experiments were performed by illumination by a single optical vortex pulse from this laser source. Temporal evolution of the ejected donor droplet was observed using a high-speed camera with a frame rate of 1 × 10^6^ frames/second (fps).

Figure 2(B) shows the temporal evolution of a single microdroplet, ejected from the donor liquid film, which was recorded by the high-speed camera. The donor liquid film formed a blister at the location of the dark spot of the irradiating optical vortex beam, and a pL-scale, single microdroplet was ejected from the tip of the blister due to Plateau–Rayleigh instability. The diameter of the microdroplet was ca. 25 µm (volume ∼8 pL, with the microdroplet containing ca. 2.3 × 10^4^ dielectric particles on average), and it was projected and printed on a glass receiver substrate located 0.6 mm away from the donor film, forming a dot with a well-defined ring structure and an outer diameter of ca. 60 μm. The effect of pulse energy (fluence) of the irradiating laser beam was examined. When the energy was lower than ca. 150 µJ, no microdroplets were ejected, whereas at an energy higher than ca. 170 µJ, multiple droplets were ejected together to form the satellites. It is should be noted that single droplet ejection is allowed when the Ohnesorge number (Oh), determined by the dynamic viscosity, the density, the surface tension, and the droplet diameter of the liquid, is within 0.1–1. Throughout the experiments, the laser was maintained at a fixed fluence of ca. 160 µJ (ca. 8 J/cm^2^).

## 3 Experiments

**Figure 3:**
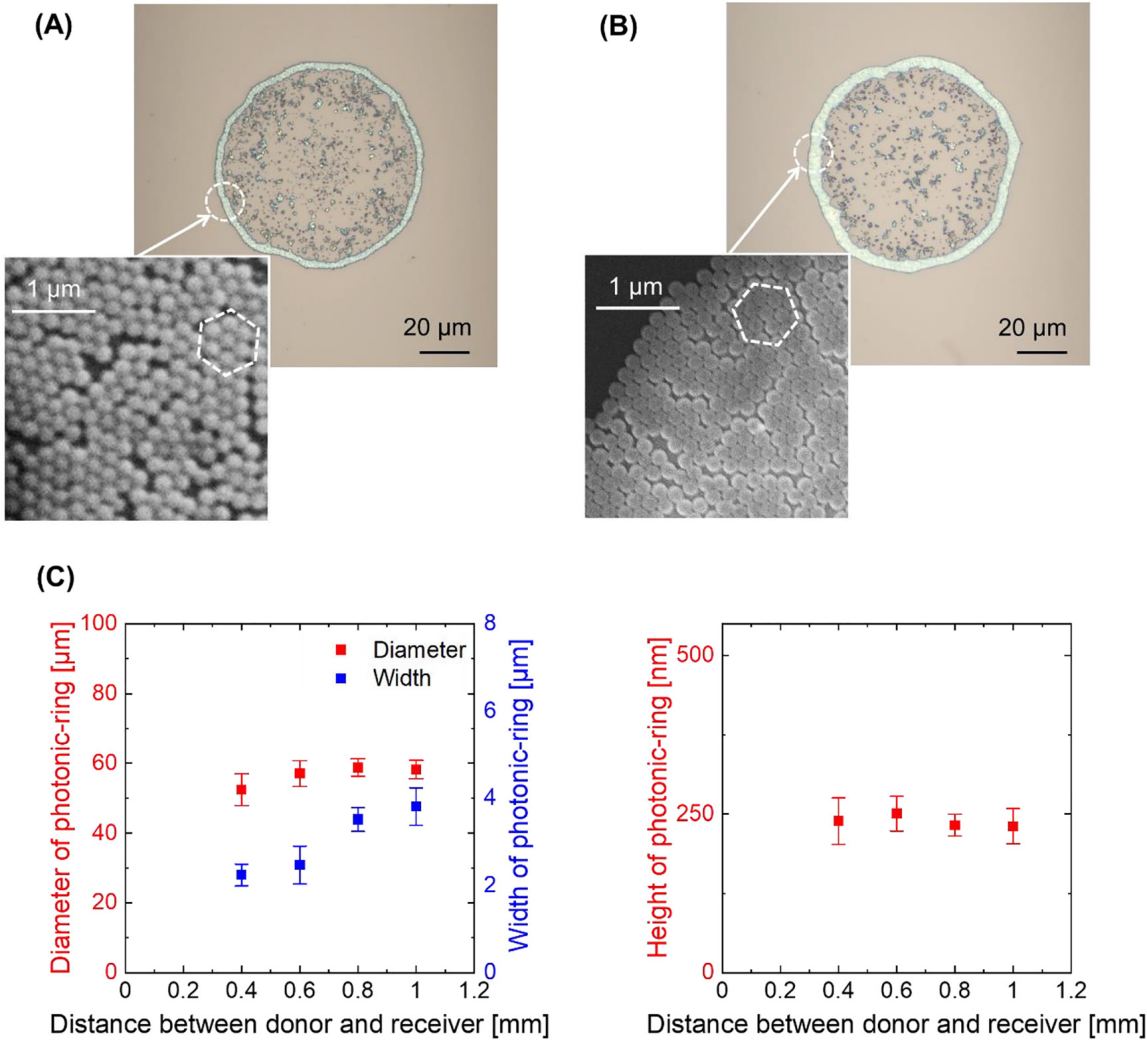
(A) Fabricated photonic-ring at a distance between donor and receiver of 0.6 mm. (B) Fabricated photonic-ring at a distance between donor and receiver of 1 mm. The fabricated rings exhibit a hexagonal closed-pack and monolayered structure (this packing structure is outlined by white-dotted hexagons in the close-up scanning electron microscope images). (C) Diameter, width and height of the fabricated photonic-rings versus distance between the donor and receiver. The height of the ring is measured to be ca. 230 nm at all positions across the structure, indicating that the ring is monolayered.

Figure 3(A) shows optical microscope and electron scanning microscope (SEM) images of a printed dot. Interestingly, a hexagonal close-packed, monolayered (ca. 230 nm thick) and ring-shaped structure (a photonic-ring) composed of the dielectric nanoparticles was formed, the shape and size of which reflects the ring-shaped spatial form of the irradiating optical vortex beam. When the distance between the donor film and receiver substrate was extended to 1 mm, the fabricated photonic-ring exhibited a nearly perfect hexagonal close-packed structure as shown in Figure 3(B). Note that this hexagonal close-packing has less free-space than square and other close-packing arrangements, and most commonly manifests as a stable 2-dimensional structure.

Monolayered photonic-rings with a thicknesses of ca. 230 nm were formed for distances >0.4 mm between the donor film and receiver, and the diameter and width of these rings were in the ranges 58–60 µm and 2.7–4.0 µm, respectively. A plot of the diameter and width of the fabricated photonic-ring as a function of donor film and receiver separation is shown in Figure 3(C). This indicates that the fabricated ring contains approximately 62% of the polystyrene nanoparticles (ca. 1.44 × 10^4^ nanoparticles), the remainder of which are scattered within the center of the photonic-ring. For a donor film to receiver separations of ≤0.4 mm, the closed-packed structure was not established well due to insufficient migration of the nanoparticles. In our experiments, the distance between the donor film and receiver was consequently fixed at 0.6 mm.

**Figure 4:**
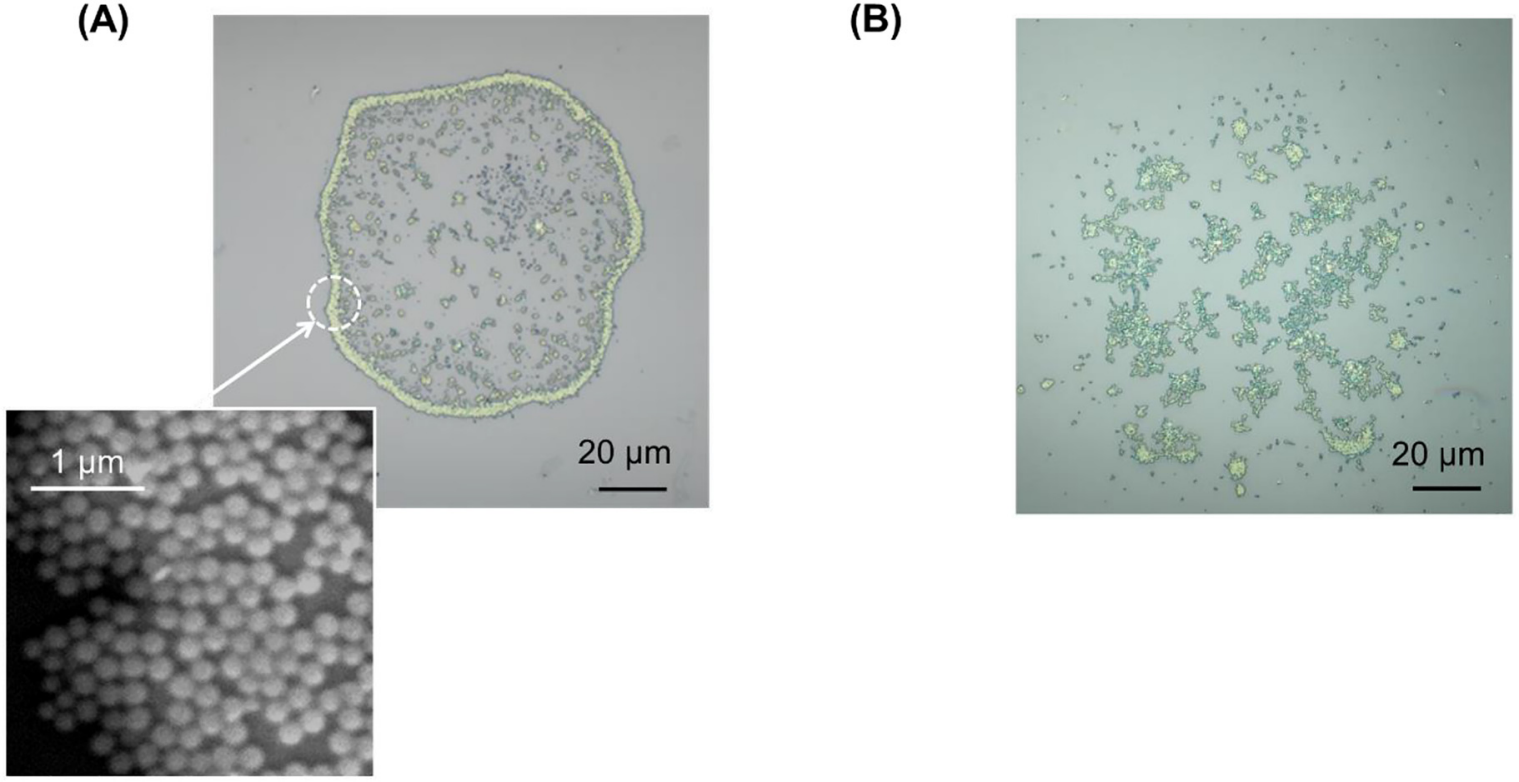
Examples of structures comprising dielectric nanoparticles formed via irradiation by (A) a radially polarized beam with a ring-shaped spatial profile; and (B) a Gaussian beam.

It should be noted that photonic-rings have never been formed by employing an axisymmetrically polarized beam with a ring-shaped spatial form (without OAM) or with the use of a conventional Gaussian beam. In fact, an axisymmetrically polarized beam has only been shown to form a nonuniform ring devoid of close-packed structures such as that shown in Figure 4(A). Gaussian beams have only produced a dot with nonuniform and nonperiodic aggregations of dielectric nanoparticles such as that shown in Figure 4(B). These characteristics show that the mechanism of optical vortex-induced photonic-ring formation is completely different from the conventional ‘coffee ring effect’ which arises as a result of Marangoni flow.

**Figure 5:**
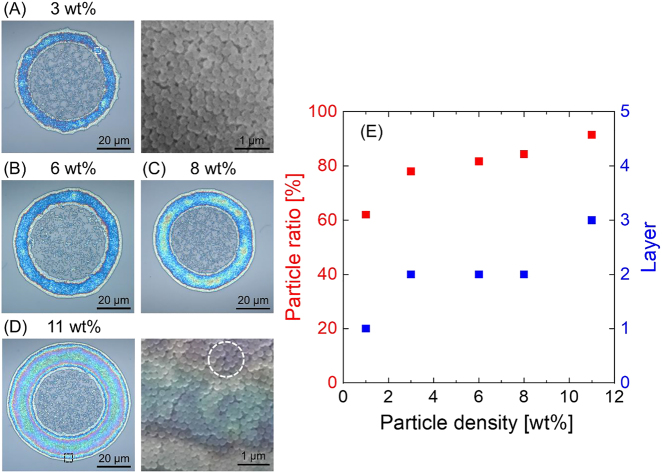
Fabricated photonic-rings formed using donor layers with particle densities of (A) 3 wt%, (B) 6 wt%, (C) 8 wt%, and (D) 10 wt%. (A)–(D) exhibit a blue structural color owing to their close-packed multiple-layered structures (indicated by the dotted hexagons in the SEM images). (A) and (D) also include SEM images of the photonic-rings. Also, note that optical and SEM images are superposed in (D). The red color, which manifests in some areas of the photonic rings, arises from ‘loosely’ close-packed structure (shown as the dotted circle). (E) Plot of the particle ratio (the number of particles in the ring divided by the total number of particles in the printed dot) versus particle density. The fabricated rings include 78–92% of all the nanoparticles contained in the microdroplets; the number of layers are also indicated.

Multilayered photonic-rings were formed when the optical vortex beam was incident on donor films with high particle densities of 3–11 wt%, as shown in Figure 5(A)–(D). Here, the fluence of the irradiating optical vortex beam was the same (8 J/cm^2^) as that in the aforementioned experiments. The multi-layered, hexagonal close-packed nanoparticles form a periodic structure which exhibits photonic band gap characteristics. As a result, the structure exhibits ‘structural color’ with a diffraction wavelength *λ* which is estimated by the expression,

(1) $$\lambda =\sqrt{\frac{8}{3}d^{2}\left(n^{2}-1\right)}\text{,}$$

where *d* is the particle diameter and *n* is the refractive index, respectively [25]. By substituting experimental parameters into the expression, the ‘structural color’ wavelength *λ* of the formed structures was estimated to be ca. 464 nm. The fabricated photonic-rings as shown in Figure 5(A)–(D) exhibited blue structural color when illuminated under white light, owing to their nearly perfect hexagonal close-packed structure (Figure 5(A)). The structure formed using a higher-density of particles (11 wt%) produced a red-rimmed photonic-ring and this is attributed to the structure being a ‘loosely’ close-packed structure (Figure 5(D)). As detailed in Figure 5(E), a very high percentage (78–92%) of the nanoparticles were contained within the fabricated ring structure with the remainder scattered within the center of the ring structure. The formation of these structures indicate that the optical attractive force of the optical vortex works to trap the dielectric nanoparticles with a high density within the irradiating optical vortex field. These trapped dielectric nanoparticles are then packed at the edge of the spinning microdroplet (due to OAM transfer effects) to form a photonic-ring inside the microdroplet.

To further investigate the effect of the optical force working on the nanoparticles, experiments were performed using a colloidal aqueous suspension of monodispersed Au nanoparticles (diameter: 150 nm, mass concentration: 64 ppm (1.84 × 10^−4^ particles/µm^3^), relative dielectric constant *ε*/*ε*_0_ ∼ −5 + 2.5*i*) in contrast to the dielectric nanoparticles (*ε*/*ε*_0_ ∼ 2.5) already detailed. The Au nanoparticle suspension was deposited on a glass substrate to form a liquid film with a thickness of 50 µm, in the same way as the experiments using the polystyrene nanoparticles. The optical density of this sample was measured to be ca. 0.74 (optical path: 10 mm). It should be noted that the ejected microdroplet from the film (on irradiation with the vortex beam) was trapped on a 1 nm-thick Au layer which was coated onto a glass substrate (this layer and substrate formed the receiver). It was observed that the residual optical vortex beam, which was not absorbed by the donor film, partially ablated the Au/glass receiver. This ablated region took the form of a ring (i.e. a ring of glass substrate was exposed where the 1 nm Au film was ablated) and this was used to accurately locate the position of the Au nanoparticles projected from the film.

**Figure 6:**
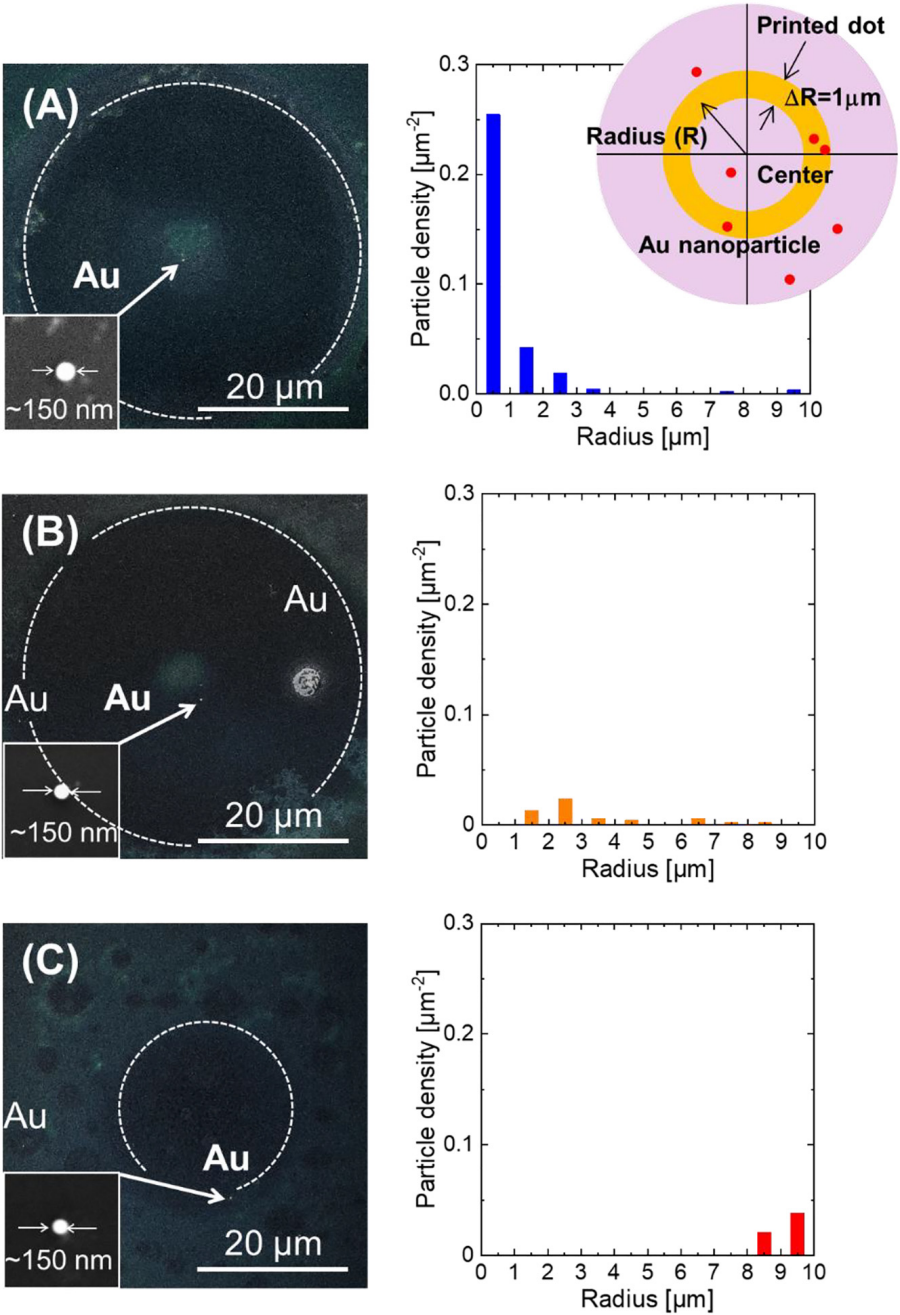
(A) A plasmonic Au nanoparticle (SEM image) in a printed dot by irradiation with an optical vortex beam. The optical vortex traps the single Au nanoparticle to form the core of the printed dot. Particle density is also defined as a number of particles being within a defined orange zone (at a distance *R* from the center of the printed dot) divided by the orange zone area. The particle density are averages of 10 replicates. Plasmonic Au nanoparticles in a printed dot by irradiation with a (B) radially polarized beam with a ring spatial form (no OAM), and (C) a Gaussian beam. Dotted circles show outer edges of irradiated optical vortex, radially polarized and Gaussian beams.

Interestingly, for a separation of >0.6 mm between the donor film and receiver, a single Au nanoparticle was deposited at the center of the ablated spot and there was no evidence of the Au nanoparticles being deposited in a ring formation. This single deposited Au nanoparticle deposited within the spot, is herein referred to as a plasmonic-nanocore; this is shown in Figure 6(A). It should be also noted that the nanocore is typically found within 1 µm of the center of the ablated spot without any undesired satellite nanoparticles. A statistical analysis of the number and position of deposited nanocores was performed. Here, a density of particles, defined as a number of particles being within an orange zone (at a distance *R* from the center of the printed dot) divided by the orange zone area 2*πR*·Δ*R* (insertion view in Figure 6(A)) was measured across 10 replicates. It was observed that the nanocore was more than 10 times likely to be positioned within 1 µm from the center of the dark spot than regions >2 µm from the center.

The formation of the plasmonic-nanocore was a characteristic unique to illumination of the donor film with a vortex beam and it did not occur when illuminating the donor film with an axisymmetrically polarized beam with a ring-shaped spatial profile (a beam without OAM), or when illuminating with a conventional Gaussian beam. Figure 6(B) and (C) show SEM images of the dots ablated by the axisymmetrically polarized and the Gaussian beams. The axisymmetrically polarized beam has only deposited Au nanoparticles significantly off center of the dot (the location of the nanoparticle was within 3–6 µm from the center of the dark spot). The Gaussian beam produced only an ablated dot (typical diameter of ca. 60 µm) with a nonuniform distribution of Au nanoparticles at its edge. Similar statistical analysis on the deposition of the Au nanoparticles was also performed under these two illumination schemes.

**Figure 7:**
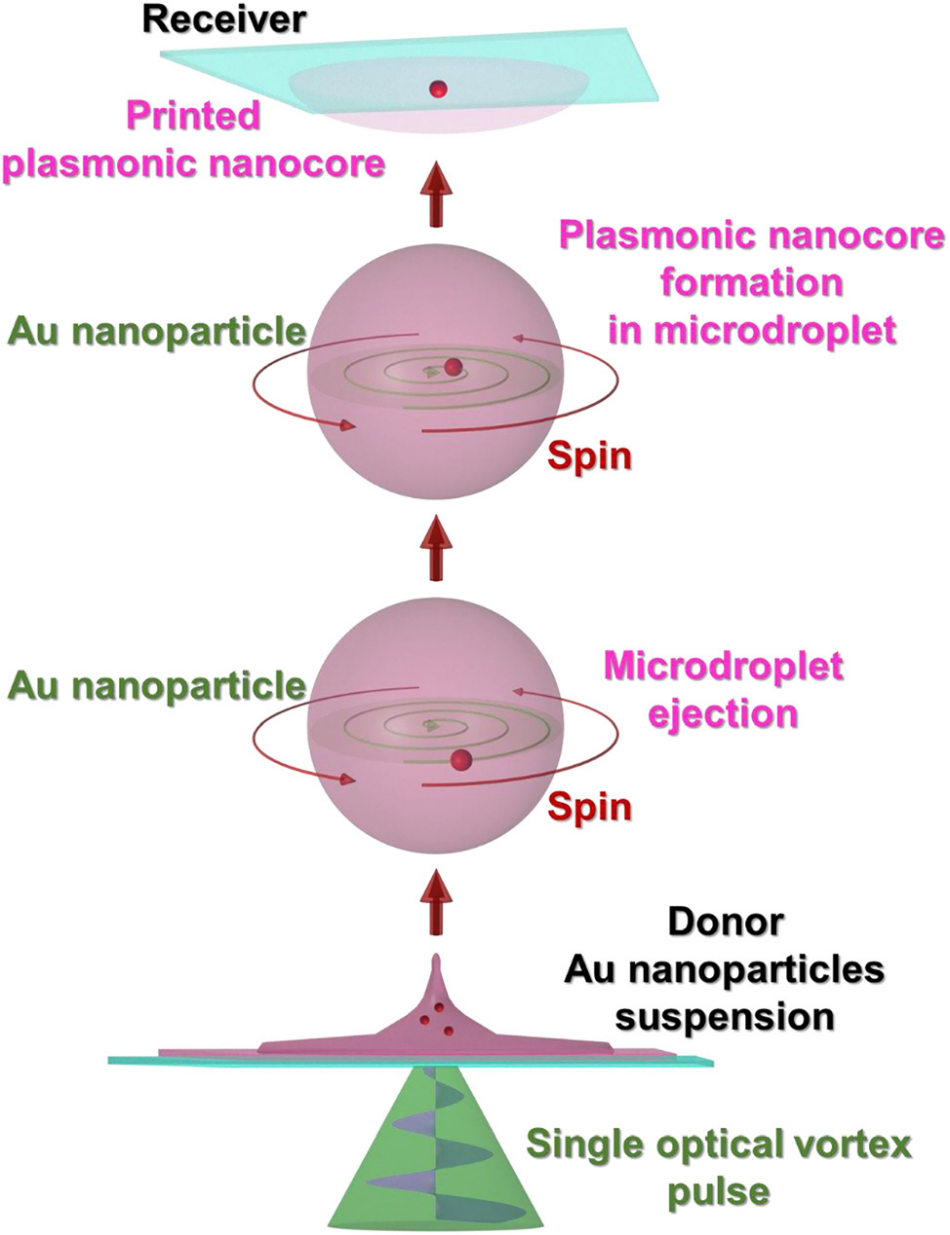
Schematic diagram of the direct print of a plasmonic nanocore by illumination by a single optical vortex pulse.

These results indicate that the optical vortex traps only a single Au nanoparticle from the donor film within its dark core, and pushes additional Au particles toward its edge by repulsive force via plasmonic resonance [5] with the aid of OAM (Figure 7).

## 4 Discussion

The optical forces operating on a nanoparticle can be qualitatively understood by examining the gradient force imparted by the laser field. The gradient force **F**_**g**_ is given by

(2) $$F_{g}\left(r\right)=\frac{1}{2}\alpha_{\text{R}}\nabla I\left(r\right)\text{,}$$

(3) $$I\left(r\right)\propto r^{2}{\text{e}}^{\frac{-2r^{2}}{\omega_{0}^{2}}}\text{,}$$

where *α*_R_ is the real part of the polarizability, *r* is the coordinate in the radial direction, and *ω*_0_ is the mode field radius (ca. 25 μm) of the optical vortex. Thus, the gradient force should attract dielectric nanoparticles with a positive *α*_R_. It should also be noted that in the case of metallic nanoparticles (with a negative *α*_R_), repulsive plasmonic forces may also be induced depending on the wavelength of the illuminating laser, that is a blue-tuned laser for the plasmonic resonance will repel the metallic nanoparticles by the plasmonic repulsive gradient force.

**Figure 8:**
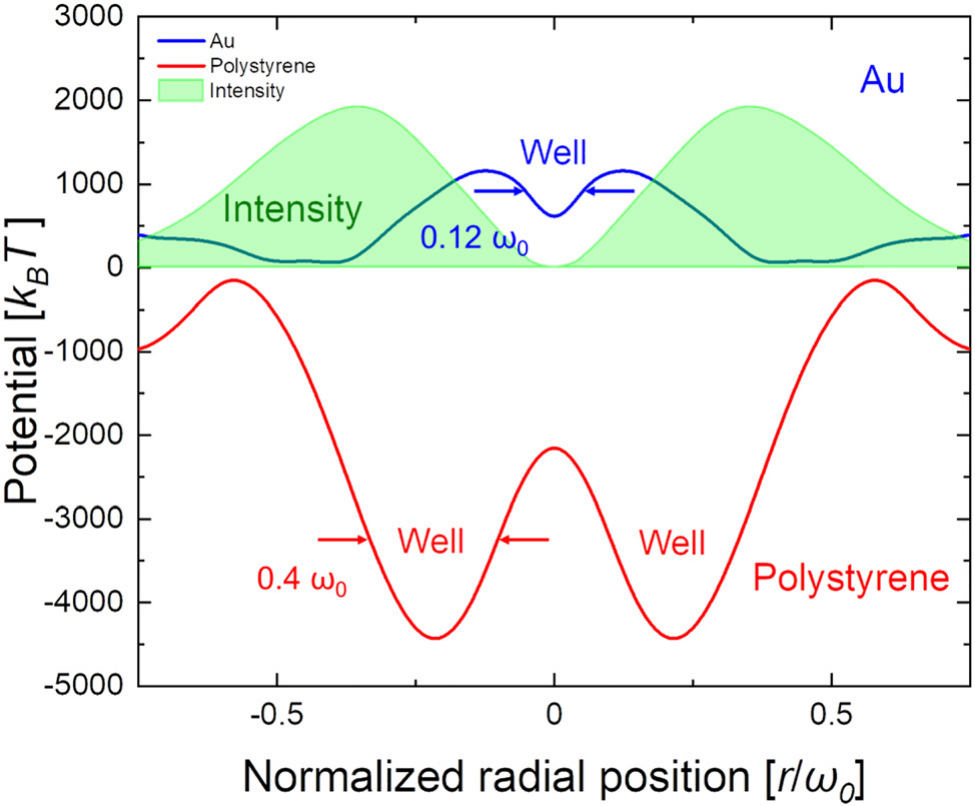
Numerically simulated optical trapping potentials produced by the irradiating optical vortex pulse. The radial optical force traps strongly the polystyrene nanoparticles within the bright ring of the optical vortex. The full width at 50% depth of the potential well for nanoparticles is estimated to be ca. 10 µm (ca. 0.4*w*_0_), which is in close agreement with the experimentally fabricated photonic-ring width (ca. 4 µm). Conversely, the repulsive optical force confines Au nanoparticles within the dark spot (ca. 0.12*w*_0_) of the irradiating optical vortex.

The optical force acting on the polystyrene and Au nanoparticles under optical vortex illumination in this work was numerically calculated by surface integration of Maxwell’s stress tensor on the nanoparticle [26, 27]. Here, we used commercial software (COMSOL). As shown in Figure 8, the radial optical force strongly traps (trapping potential depth ∼ −2260 *k*_B_*T*) the polystyrene nanoparticles within the bright ring (potential well) of the optical vortex. The full width at 50% depth of the potential well was estimated to be ca. ∼10 µm (0.4*ω*_0_), which supports the experimentally fabricated photonic-ring width (ca. 4 µm). Conversely, the plasmon-induced repulsive optical force confines Au nanoparticles within ca. 3 µm (0.12*ω*_0_) (the full width at 50% depth of the potential well) from the center of the dark spot of the irradiated optical vortex. Its potential well depth is estimated to be ∼−550 *k*_B_*T*. These numerical simulations support our experimental observations.

**Figure 9:**
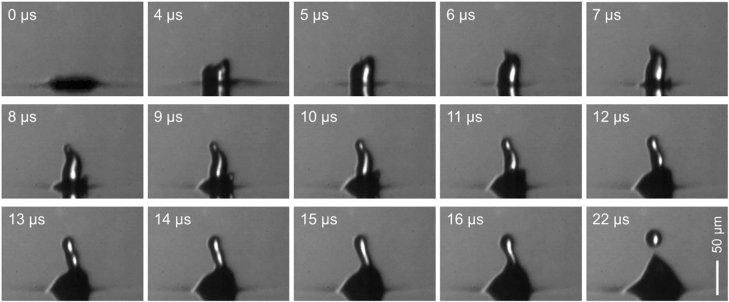
Temporal evolution of the droplet ejection by the irradiating optical vortex at a high temporal resolution (1 × 10^6^ fps). The blister appears to be twisted toward a counter-clockwise direction.

Understanding the formation of the close-packed nanoparticles within the microdroplet requires understanding of the torque acting on the microdroplet. The OAM-induced angular torque *T*_OAM_ of a microdroplet is given by the expression,

(4) $${Τ}_{\text{OAM}}\approx \frac{E}{\omega }\cdot \frac{l}{t}\text{,}$$

where *E* is the pulse energy, *t* is the pulse duration, *ω* is the angular frequency, and ℓ is the topological charge of the irradiating optical vortex beam. The Stokes drag torque *τ* of the microdroplet is given by the following expression [28]

(5) $$\tau =8\pi \sigma a^{3}\left(2\pi f\right)\text{,}$$

where *σ* is the viscosity of the surrounding air (1.8 × 10^−5^ (air) Pa·s), *a* is the radius of the sphere, and *f* is the rotation frequency, respectively. The microdroplet is here assumed to be a rotating sphere. The terminal rotation frequency *f* of the microdroplet is estimated to be ∼1 × 10^6^ rotations/second (rps), as is clear from the fact that the blister (with a rather large mass) formed by the irradiating optical vortex appears to be twisted (Figure 9, Supplementary Movie). This estimation comes from the fact that we could not directly observe the ultrafast spinning motion of the droplet due to insufficient temporal and spatial resolution (2 μm) of the high-speed camera. It is therefore difficult to quantitatively explain how OAM assists the packing/confinement of the nanoparticle at this stage.

To fully understand the mechanism of this structuring within the microdroplet, direct observation and numerical analysis of the spinning motion of the microdroplet based on hydrodynamics [29, 30] must be further studied. Furthermore, we believe that the difference in OAM-induced forces on dielectric and metallic nanoparticles will yield a novel sorting method in which dielectric and metallic nanoparticles in a mixture can be spatially separated using optical vortex-induced forward transfer (‘optical vortex nanoparticle-sorting’). This will be examined in our future work.

## 5 Conclusions

We have demonstrated the direct-print of a hexagonal close-packed, monolayered/multilayered and ring-shaped structure formed of dielectric nanoparticles on a substrate via optical vortex-induced forward transfer. We have also demonstrated the direct-print of a single Au plasmonic-nanocore with a high spatial resolution, through the application of plasmonic-resonant repulsive force of an irradiating optical vortex beam. The formation of these exotic structures results from the unique attractive and repulsive forces which manifest between the OAM of the optical vortex beam and different materials. Indeed, we believe the unique manner, in which the OAM interacts with dielectric and metallic materials can also be used as a means of sorting mixtures of nanoparticles.

We anticipate that the application of vortex-induced forward transfer techniques will pave the way toward advanced cost- and time-saving, noncontact nano-/micro-fabrication technologies with a long (millimeter-scale) working distance. Such technologies which will be pivotal for the next generation of printed photonic/plasmonic devices including microring lasers [31], [32], [33] and plasmonic antennas.

## Supplementary Material

Supplementary Material

Supplementary Material

## Supplementary Material

Temporal evolution of the droplet ejection by the irradiating optical vortex at a high temporal resolution (10^7^ fps). The blister appears to be twisted toward a counter-clockwise direction.
